# The association between social support and antenatal depressive and anxiety symptoms among Australian women

**DOI:** 10.1192/j.eurpsy.2022.551

**Published:** 2022-09-01

**Authors:** A. Tilahune, W. Peng, J. Adams, D. Sibbritt

**Affiliations:** University of Technology Sydney, Public Health, Sydney, Australia

**Keywords:** Pregnancy, social support, anxiety symptoms, depressive symptoms

## Abstract

**Introduction:**

Antenatal depression and antenatal anxiety adversely affect several obstetric and foetal outcomes, and increase the rate of postnatal mental illness. Thus, to tackle these challenges the need for social support during pregnancy is vital.

**Objectives:**

This study examined the association between domains of social support and antenatal depressive and anxiety symptoms among Australian women.

**Methods:**

Our study used data obtained from the 1973–78 cohort of the Australian Longitudinal Study on Women’s Health (ALSWH), focusing upon women who reported being pregnant (n=493). Depression and anxiety were assessed using the Center for Epidemiological Studies Depression (CES-D-10) scale, and the 9-item Goldberg Anxiety and Depression scale (GADS) respectively. The 19 item-Medical Outcomes Study Social Support index (MOSS) was used to assess social support. A binary logistic regression model was used to examine the associations between domains of social support and antenatal depressive and anxiety symptoms.

**Results:**

After adjusting for potential confounders, our study found that the odds of antenatal depressive symptoms was about four and threefold higher among pregnant women who reported low emotional/informational support (AOR=4.75; 95% CI: 1.45, 15.66; p=0.010) and low social support (overall support) (AOR: 3.26, 95%CI: 1.05, 10.10, p=0.040) respectively compared with their counterpart. In addition, the odds of antenatal anxiety symptoms was seven times higher among pregnant women who reported low affectionate support/positive social interaction (AOR=7.43; 95%CI: 1.75, 31.55; p=0.006).

**Conclusions:**

Low emotional support and low affectionate support have a significant association with antenatal depressive and anxiety symptoms respectively. As such, targeted screening of expectant women for social support is essential.

**Disclosure:**

No significant relationships.

